# The Accuracy of CT Scanning in the Assessment of the Internal and External Qualitative Features of Wood Logs

**DOI:** 10.3390/s23208505

**Published:** 2023-10-16

**Authors:** Miloš Gejdoš, Tomáš Gergeľ, Katarína Michajlová, Tomáš Bucha, Radovan Gracovský

**Affiliations:** 1 Department of Forest Harvesting, Logistics and Ameliorations, Faculty of Forestry, Technical University in Zvolen, T. G. Masaryka 24, 960 01 Zvolen, Slovakia; gejdos@tuzvo.sk (M.G.); xmichajlova@is.tuzvo.sk (K.M.); 2 National Forest Centre, Forest Research Institute, T. G. Masaryka 22, 960 01 Zvolen, Slovakia; tomas.bucha@nlcsk.org (T.B.); radovan.gracovsky@nlcsk.org (R.G.)

**Keywords:** CT-scanner, qualitative features of wood, qualitative evaluation, quality determination, raw-wood assortments

## Abstract

The qualitative evaluation of harvested raw logs and sawlogs is mainly based on the quantitative and qualitative evaluation of the visible macroscopic features of the wood. Modern methods allow for the analysis of whole logs by means of computed tomography. These devices can analyze the internal qualitative features of wood that are not visible on the external structures of the logs. The aim of this work was to evaluate the detection accuracy of a CT-scanning device intended for scanning logs on the internal qualitative features of wood using model trunks. Two logs of beech and oak with a length of 4 m were selected for the analysis, based on availability. Qualitative features were identified through computed tomography scanning, visually identified on cut sections, and then manually measured in accordance with applicable legislation. Relatively good agreement was demonstrated for the detected features in terms of identifying their location (dimension in millimeters from the end of the log). For this parameter, the average differences were 0.90% on the beech log and only 1.21% on the oak log. Relatively high accuracy was shown via CT detection of qualitative features in the beech section (with average differences in dimensions of only 3.5%). In the case of the oak log, the dimensions of the quality features were significantly overestimated. These results indicate that CT scanning technology may have a problem with some hardwood species. It was primarily developed for coniferous tree species, and software algorithms are, therefore, not yet fully adapted to the precise detection of the dimensions of individual quality features. Despite the detected differences, it was confirmed that the CT technology of scanning harvested wood can have a fundamental impact on optimization procedures in the recovery and processing of wood. Renting a scanning line for a certain capacity of wood volume appears to be a deployment option for forestry operations and smaller wood processing operations. Thus, this technology can become an important factor in improving the economic evaluation of the final production of wood.

## 1. Introduction

With the dynamic development of information and communication technologies, there has been progress in digitization processes in the field of forestry as well. Current technologies and mathematical–statistical dendrometric methods, with the development of the mobility of terrestrial laser scanning, enable the relatively accurate quantification of the volume of wood in forest stands [1,2,3]. Obtaining information about the qualitative structure of trees in a forest stand is mostly based on a system of quantitative and qualitative features and is largely influenced by the subjectivity of the evaluator [4,5,6,7]. For determining the internal structure of standing trees, there is a relatively large number of methods that are based on destruction, semi-destruction, or computer tomography—specifically, acoustic tomography [8,9,10,11]. The spectrum of these methods allows for the relatively simple and relatively accurate identification of internal damage; however, in most cases, these methods are only limited to the ground part of the trunk.

The qualitative assessment of harvested raw logs and logs is also based on a quantitative and qualitative assessment of visible macroscopic features. In the case of hidden qualitative features in larger volumes of wood, available evaluation methods are relatively limited [6,12,13]. Modern methods allow for the analysis of whole trunks by means of computed tomography. These devices can analyze the internal qualitative features of wood that are not visible on the external structures of the logs. The problem is the too narrow portfolio of these devices and, at the same time, their high acquisition costs. There are currently 14 such CT lines installed in the world, which are located primarily in wood processing industries where they enable the optimization of cutting and processing plans for raw-wood assortments [14,15,16].

Through the combination of X-ray projections obtained from different directions, tomographic reconstruction allows for the obtaining of a different value of X-ray absorption for each point of the measured space. Based on the measured value of radiation absorption depending on the density of a given point in the tested body, this point is assigned a value of gray color as well. Instead of a 2D image divided into rectangles called pixels, we obtain a 3D image divided into parallelograms called voxels [17]. The anatomical composition of wood, especially carbon chains, allows the results of computed tomography to be very similar to those obtained in medicine when analyzing the internal parts of the human body. The first tests were performed on strains in the early eighties [18,19]. There have been a number of studies focused on automatic CT image analysis and optimization (e.g., [20]), and a database of CT scan records was created in Sweden [21]. In the 1990s, state-of-the-art sawmills began using static X-ray scanners to detect the internal features of logs. X-ray scanners are usually based on discrete X-ray scanning in 1–4 directions, with the protocol fed through the scanner [22,23]. Discrete X-ray scanners provide information on properties such as heartwood proportion, strength, density, knot size and volume, whorl spacing, and tree-ring width [24,25,26].

In 2008, the development of a CT scanner with the characteristics required for an industrial process and the ability to optimize the cutting of individual logs based on their internal features began [27,28].

The importance of the possibilities of deploying these innovative technologies in forestry operations is based on forecasts of the future development of raw-wood assortments prices [29] and requirements to ensure the sustainability of the industry [30].

The aim of this work was to evaluate the detection accuracy of a CT-scanning device intended for scanning logs on the internal features of wood using model tree trunks. Accuracy was evaluated on the basis of comparative analysis with visual evaluation and measurement of internal qualitative features, which were subsequently evaluated from cut-up and scanned logs.

The wood processing industry evaluates the qualitative characteristics and quality of wood differently, especially from the point of view of the finalization of its products compared to forestry operations. Incorrect storage and trading methods often lead to the damage and deterioration of the raw material of wood. The cause is mostly biotic harmful factors in combination with abiotic factors (e.g., temperature + moisture—an indication of increased damage by fungi). The characteristics and assessment of this damage are discussed, e.g., in work [31]. In the recent past, there have been several research initiatives focused on the issue of assessing the quality of wood and wood products (e.g., COST Action E53). Even though the importance of coordinated research in this area has been demonstrated, until now there has been a lack of an integrated specialized workplace that could engage with this issue in a complex way [32].

## 2. Materials and Methods

Two logs were selected for analysis based on availability. The first log was beech, and the second log was oak. Both logs were 4 m long at the time of scanning. Since part of the oak log was used in another analysis, only its initial two-meter part was evaluated in the comparative analysis. The entire four-meter section of the beech log was evaluated.

### 2.1. Log CT Scanning Methodology

**Figure 1:**
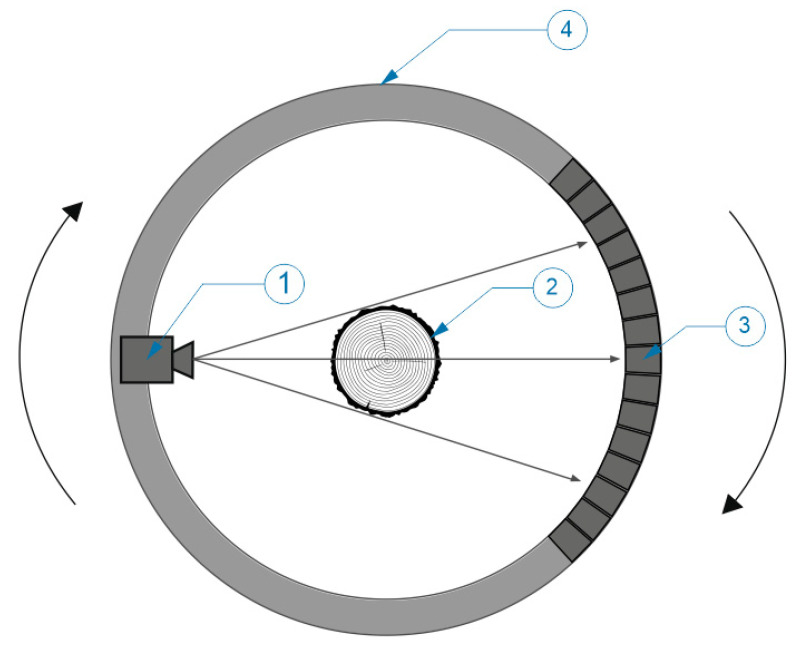
Schematic representation of CT scanning principle 1—X-ray source, 2—log, 3—detector field, 4—rotation ring.

The scanned trunk passed inside the scanner using a conveyor, where it was exposed to X-ray radiation (Figure 1). The feature of the absorption of X-ray radiation by wood and its specific qualitative features was used to display the internal structure. Each scanned slice of the trunk was imaged using a tomographic inversion algorithm. The software processed the output from the scanning algorithm in the form of a digital image (*.tiff format), which contained individual sections of the trunk. The individual steps of these cuts were at distances of 10 mm. For each centimeter of length, there was 1 scanned section of the trunk. The entire 3D model of the scanned trunk could then be reconstructed from these images in the software. Virtual cutting plans could be made from this model in all directions.

The scanner had limitations in terms of pixel size (10 mm in the longitudinal direction and 2 × 2 mm in the transverse direction). The absorption of X-ray radiation within the scanned trunk was expressed by the brightness value of the pixels in degrees of gray (DN value). If the density of the material was lower, then the pixels were displayed as a darker shade of gray. If the density of the material was higher, then the pixels were displayed as a lighter shade of gray. Changes in wood density were also visible, depending on the structure of annual rings and the presence of qualitative features (knots, cracks, rot).

**Figure 2:**
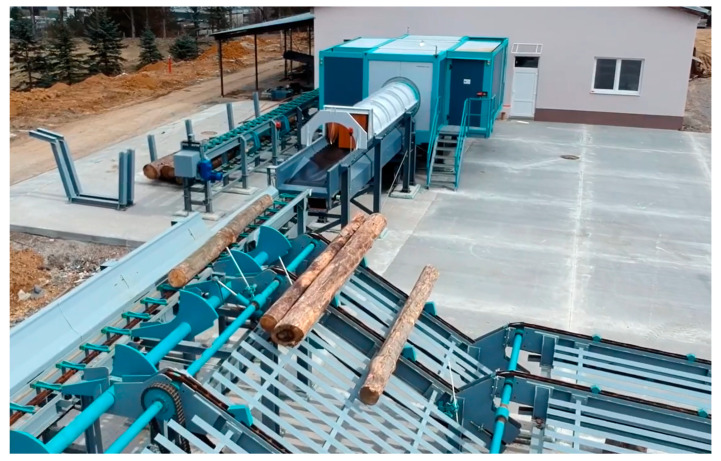
CT LOG X-ray Computer Tomography scanner—National Forest Center Zvolen—Biotechnological Park Stráž.

A CT LOG X-ray Computer Tomography scanner from Microtec was used for evaluation analysis (Figure 2). The scanner is mounted on a static frame supporting a rotating gantry using a large cone beam and reconstruction algorithm. The gantry is equipped with a fully shielded and water-cooled X-ray source and a multiple-line sensor ring. The scanner is equipped with these evaluation software: CT IO (version 1.0.15) + CT Recon; CT Pro and CT Viewer (version 3.05). The CT IO system manages the CT Log hardware (scanner) and, together with the CT Recon system, also pre-processes the raw data coming from the X-ray sensors. CT Pro software receives input from CT Recon, analyzes it, and identifies a list of features and properties of the scanned log. CT Viewer (version 3.05) receives inputs from CT Pro. It allows for the visual evaluation of logs and the simulation of cutting plans in 2D or 3D environments.

The tested pieces of the logs were tomographically scanned using this method. Various 3D models of the tested logs were created in specialized software for processing CT images—3D Slicer (freeware, version 4.11.20210226 r29738/7a593c8). A cutting plan was virtually realized on these models, which was identical to the real cutting plan realized on a log band saw. Subsequently, virtual pieces of sawn wood identical to the real saw wood were created. As with real saw wood, the individual qualitative features of the wood were identified and marked. Using the built-in measuring function of the 3D Slicer software (version 4.11.20210226 r29738/7a593c8), the distance of the feature from the top end of the log was recorded, and in accordance with the method in the STN EN 1309-3 standard, the dimensions of each feature were measured. The resulting values were entered into a comparison table together with the actual values measured on real pieces of saw wood, which were created by implementing the log-cutting plan.

### 2.2. Exclusion of the “Ring Artifact”

**Figure 3:**
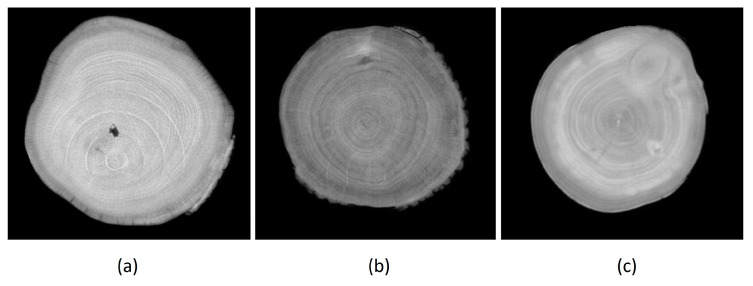
Ring artifact depending on the thickness of the log (**a**) log with a thickness of over 650 mm; (**b**) log with a thickness of 600 mm;(**c**) log with a thickness of 450 mm.

Our experience has shown that when scanning rough logs, a so-called “ring artifact” (Figure 3a) occurs on the processed images, which could affect the scanning results. However, through our praxis and use of the scanner, we have come to the conclusion that this phenomenon is visibly demonstrated only in logs whose thickness is greater than 650 mm (Figure 3a). With logs with a thickness of 600 mm, it is only slightly noticeable (Figure 3b), and with logs with a normal thickness of 400 mm and less, this phenomenon disappears (Figure 3c). This is due to the settings of the processing software algorithms of the scanner, which can filter out this phenomenon. If thicker slices than the recommended operating thickness of the scanner are scanned, this phenomenon is visible. Since none of the measured logs reached the thickness parameters of more than 450 mm, this phenomenon could not fundamentally affect the image analysis and the subsequent results of the comparison.

### 2.3. Methodology of the Manual Measurement of Qualitative Features

**Figure 4:**
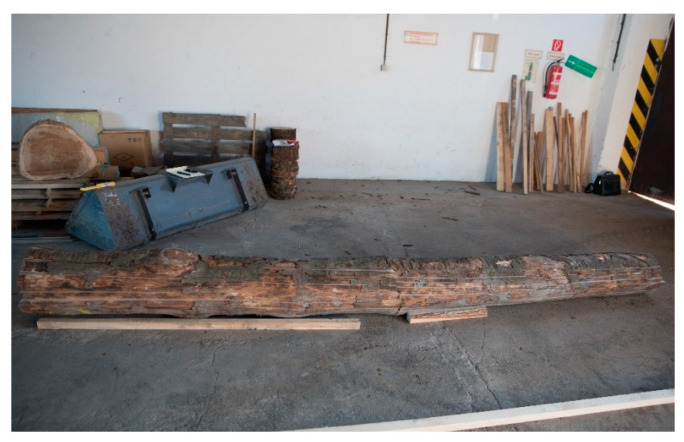
A beech log was cut and arranged again in the whole log.

**Figure 5:**
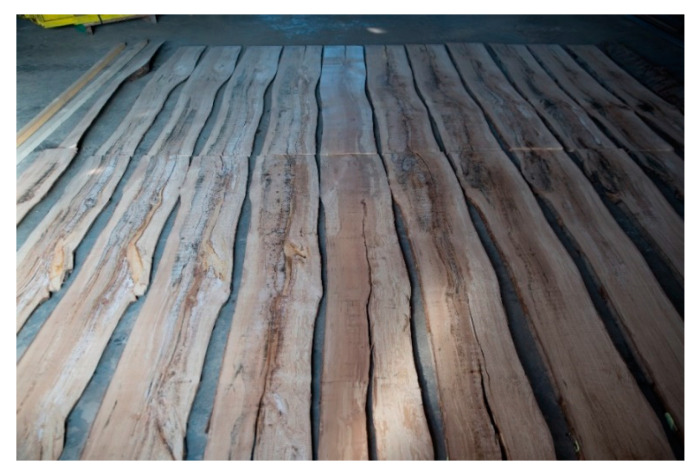
Unfolded sections from a beech log in the order in which they were arranged throughout the log.

**Figure 6:**
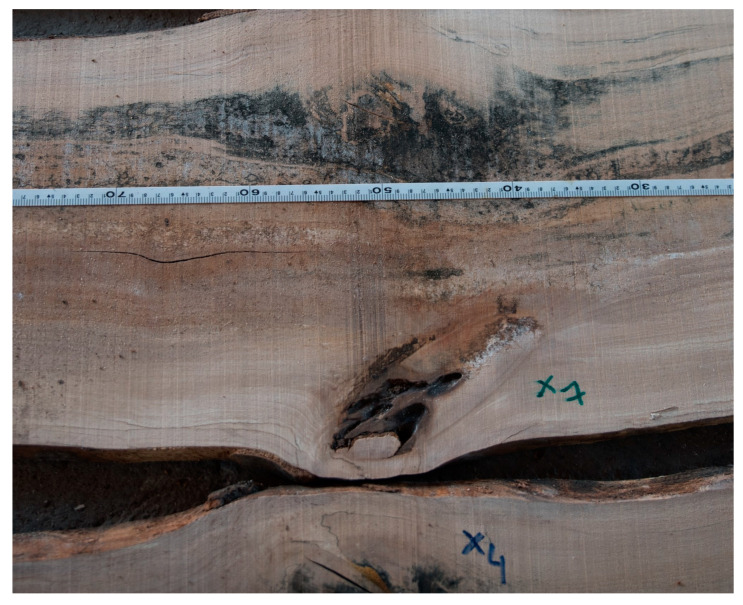
Identification, labeling, and measurement of internal qualitative features on sections from the beech log.

The scanned logs were longitudinally cut with a log band saw Mebor HTZ 1000 into sections 2 cm thick, which were then laid out in the order as they were arranged in the whole log (Figure 4 and Figure 5). The thickness of the saw blade was 4 mm. A simple cutting plan was implemented. The thickness of the slices was set at 20 mm. The first surface of the cut was measured at a thickness of 20 + 4 mm. The 3D slicer allowed virtual slices to be moved with a step of 1 mm. A visual analysis of the trunk surface’s qualitative features was performed on the composite log. Identified features were subsequently measured in accordance with the standard STN EN 1309-3 [33]. After the layout of the individual cutouts, qualitative features were visually identified and subsequently marked, which were then manually measured again in accordance with the STN EN 1309-3 standard (Figure 6).

STATISTICA 12.0 software was used for basic statistical analyses, and statistical parameters were calculated according to generally known scientific calculation procedures.

## 3. Results

**Figure 7:**
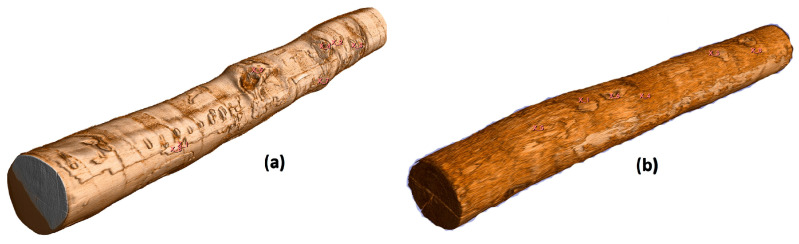
A 3D reconstruction of the logs (**a**) beech and (**b**) oak from the scanning outputs of the CT tomograph, also with the identification of qualitative features on the round surface of the logs.

**Figure 8:**
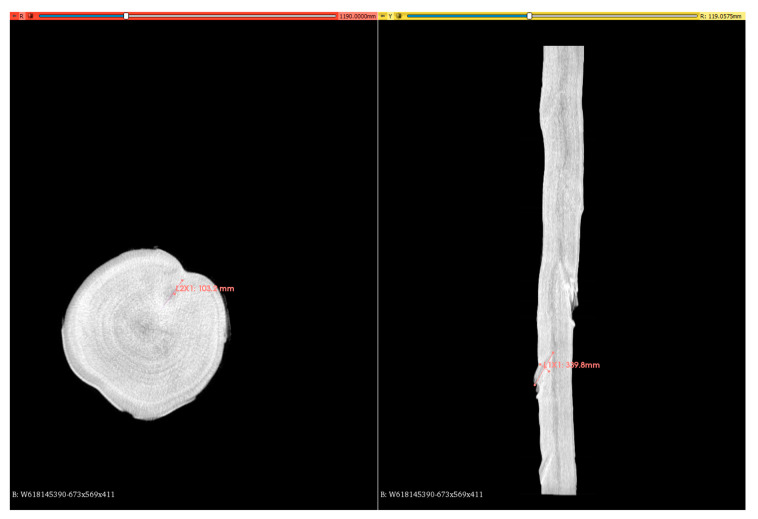
Analysis of internal qualitative features of the beech log using scans of the internal sections of the log.

Reconstruction of the 3D models of the analyzed logs, including the identification of surface qualitative features in the 3D slicer software environment, is shown in Figure 7. The identification of the internal qualitative features of the wood was performed from scans of individual cuts of the log (Figure 8).

The results of the identification of qualitative features based on visual assessment and measurement in accordance with the STN EN 1309-3 standard together with the identification from the scanned outputs of the CT scanner are shown in Table 1 and Table 2. From the point of view of practical applicability and verification of the accuracy of CT scanning, only internal qualitative features were dimensionally evaluated.

The majority of quality features did not represent a limiting factor for the qualitative classification of the log into a specific quality class. It should also be noted that approximately 1 month passed from the time of scanning the section to its visual assessment and manual measurement. Even though the research was carried out in the winter, slight color changes of the conductive meshes and the beginning phase of mold were evident, especially on the beech log. However, all of the research was conducted in the winter months, and the trunks that were scanned were not freshly harvested, so some moisture loss had already occurred in the process prior to scanning. They were then stored in a cool, dark room. However, the natural dimensional changes were not of a fundamental nature. These features were, therefore, not taken into account during visual evaluation. Overall, the CT scanner did not identify a false heartwood feature on the beech log and one knot on the oak section.

Table 3 evaluates the differences between individual features detected by manual measurement and digital measurement from CT scanning. The differences were evaluated in millimeters, and the percentage difference between the manual measurement and the digital measurement from the CT scans was then calculated. The measured dimensions of individual quality features were evaluated in accordance with the above-mentioned standard. The classification of qualitative features was based on visual assessment.

Relatively good agreement was demonstrated for the detected features in terms of identifying their location (dimension in millimeters from the end of the log). For this parameter, the average differences were 0.90% on the beech log and only 1.21% on the oak log. Relatively high accuracy was shown via CT detection of qualitative features in the beech log (average differences in dimensions of only 3.5%). In the case of the oak log, the dimensions of the qualitative features were significantly overestimated. In the case of a bark pocket qualitative feature, it even grows more than triple, which significantly increased the average percentage difference in the dimensions of the qualitative features determined by manual and digital measurement. These results indicate that CT scanning technology may have a problem with some hardwood species. It was primarily developed for coniferous tree species, and the software algorithms are, therefore, not yet fully adapted to the precise detection of the dimensions of individual qualitative features.

The average percentage deviations significantly overestimated inaccuracies in one or two qualitative features that the software incorrectly evaluated from the CT scans. For a more accurate determination of the variability of detection accuracy, it will, therefore, be necessary to continue further research and analyze a higher number of logs and sections in order to statistically determine the deviations of CT scans from real measurements, even taking into account a specific type of wood. So far, more fundamental differences are manifested in the size of the given feature rather than in its localization in the entire log.

In total, 15 sections were identified from both trunks, on which some qualitative feature was present. The characteristics of these features were evaluated by the basic parameters of descriptive statistics listed in Table 4. These statistics speak about the variability of the dimensions of qualitative features when comparing the methodology of CT scanning and manual measurement and provide a more detailed picture of the accuracy of both methods. On average, the dimensions of the qualitative features identified differed only minimally. However, the median value was higher when the feature was detected by a CT scanner. The standard deviation indicates that the real measured values of the identified qualitative features are more widely distributed. This results in the value of the coefficient of variation. The dispersion of values was thus greater during manual measurement, which also results from the very principle of measuring characters from a digital image obtained with CT scanning.

**Figure 9:**
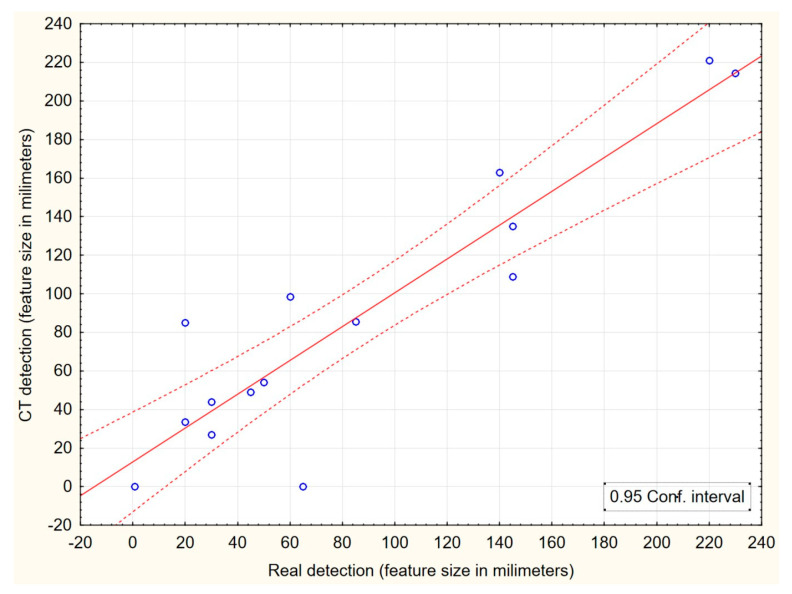
Correlation of qualitative features dimensions determined with CT detection and manual measurement at a 95% confidence interval.

In Figure 9, the mutual correlation of the identified dimensions of qualitative features through both methods (CT scanning and manual measurement) is evaluated. These results are intended to demonstrate at what size of a qualitative feature do both methods correlate as much as possible with each other and at what dimensions of the features the biggest differences occur. It is clear from the figure that the biggest differences between the two identification methods are for features up to 7 cm in size. With a larger dimension of the identified features, the mutual correlation of both methods at a 95% confidence interval increases. These results thus signal that accurate dimensional identification with the smaller dimensions of qualitative features may be the main weakness of CT detection of qualitative features. However, this does not fundamentally limit its real use to optimize cutting plans, for which this method was primarily developed. The results can also be improved with optimization updates of the evaluation software for individual tree species.

## 4. Discussion

Works that began to use computed tomography for the evaluation of the qualitative features of wood began to appear particularly after 1990. Aguilera et al. [34] 2008 compared images of knots in pine logs obtained via computed tomography and X-rays. They found that the moisture content of the wood can have a fundamental influence on the accuracy of the evaluation. When the wood is saturated with water, there are density deviations that distort images obtained with tomography. Ten logs were analyzed. However, this fundamental influence can be solved using a software algorithm. In this case, when the image is weighed by the morphology of the object, which should be identified, the system prohibits the contour from moving to places that are not justified by the nature of the object. Based on the results obtained, the authors concluded that the presented method of active contours is a good alternative in situations where the information about the objects to be segmented is known in advance. It was also confirmed in our work that the establishment of such algorithms for the evaluation software would improve the accuracy of the identification of the dimensions of the given qualitative feature.

Another work [35] evaluated the possibility of using CT stands for computed tomography for the analysis of the process of degradation of wooden materials in construction. The computed tomography method has also been shown to be of particular interest for assessing the effectiveness of structural modification strategies that are expected to inhibit decay or extend the lifetime of the material.

When comparing the methods of acoustic tomography, the resistography method, and computer tomography, which were used to analyze wooden power line poles, significant similarities in damage detection were found [36]. This confirms that less expensive evaluation methods based on acoustic tomography or semi-destructive methods based on cross-section drilling can be used for the qualitative evaluation of wood, where the exact identification of the type of qualitative feature and its exact localization is not necessary. However, such methods are insufficient for optimizing the evaluation of harvested wood and establishing optimization plans for its processing (e.g., an optimal cutting plan).

Another paper [37] established the computed tomography method as a successful technique for the analysis of damage and subsequent decomposition of wood as a result of an attack by insects. The authors developed and tested a new approach based on computed tomography scanning and a semi-automated image analysis of logs from a field experiment with manipulated bark beetle and wood-destroying insect communities. The volume of larval passages in wood and bark and the relative volume of wood showing features of fungal decay were quantified. The authors then compared both measurements with classic damage assessment approaches. The volume of insect larval passages was correlated with the loss of dry matter and clearly reflected the differences between individual species of groups of insects. Fungal decay was identified with high accuracy and strongly correlated with ergosterol content. The authors demonstrated that, even in this case, the identification of this qualitative feature is an effective approach for quantifying wood decomposition by insects and fungi.

Beaulieu et al. [38] successfully used computed tomography analysis to determine wood density based on images of white spruce (Picea glauca (Moench) Voss) sections. Virtual sections of core wood zones of three sizes (1 voxel diameter, which is the smallest volume unit on which the CT number is calculated, 5 mm, and 12 mm) were extracted in four orthogonal directions from sapwood to bark. This allowed for the evaluation of the effects of core direction and size on wood density estimates. It was found that the mean values and radial patterns of wood density estimated from the CT scan data were typical of the values and patterns reported for the white spruce species in the literature, especially in relation to the year of growth, because the experimental individuals sampled differed physiologically by age. Thus, the application of CT scanning technology in wood science enables the digital extraction of 3D data subsets to perform wood density estimation, radial sample analysis, and hypothesis testing.

Based on a literature review of existing works, it appears that the most effective method of CT image processing is an artificial neural backpropagation network [20]. Knot parameters extracted from CT images are affected by several trees or stand variables, such as silviculture regimes, thickness classes at 1.3 m heights, and knot positions in the tree trunks in the case of Scots pine [39]. On CT images, nodules usually appear lighter in tone (higher grey-level values) than the surrounding area because they are made of high-density cells. These differences in grey-level values between log features allow for the development of segmentation algorithms that use grey-level value histogram thresholds to identify areas of potential defects on CT images [20]. This fact was also confirmed in our analysis.

Studies in which CT scanning was applied with research objectives beyond the assessment of wood quality for the optimized production of forestry products emphasize the importance of analytical procedures for a graphical and quantitative analysis of CT scan data (images and numbers) and the need for the development of additional specialized algorithms and software. For example, the density of wood in which knots form is often similar to that of wet sapwood. This is why it is difficult to segment and measure knots in protocols, but several methods using a CT scanner and accompanying algorithms for data analysis and image processing have been proposed, and some show great promise for solving the problem [40]. Even the method of the evaluation of knots, which we used within the specialized software originally developed for medical purposes, confirmed this fact, and the results provide quite satisfactory accuracy in determining the dimensions of knots detected with a CT scanner.

## 5. Conclusions

Scanning technology based on a CT scanning line was primarily developed for the wood processing industry, the product of which is mainly sawn wood. This technology was primarily intended to assist in the localization of essential qualitative features that affect the quality of the produced timber. Since the investment costs for this technology range in the order of millions of euros, it was profitable only for large wood processing enterprises. Smaller processing enterprises and forestry enterprises do not have the economic means to procure and operate this technology, regardless of its indisputable benefits. Also, until now, it was not possible to procure this technology directly for research purposes.

In Slovakia, such a line was installed directly for research purposes as the first in the world under the authority of the National Forestry Center.

This work, which was based on two reference logs of non-coniferous tree species, verified the accuracy and quality of the identification of individual qualitative features based on reverse verification through visual identification and manual measurement on sections that were cut from these two logs.

It was confirmed that CT technology does not have a problem with the identification and localization of individual qualitative features in the section of the log. The differences in the localization of individual quality features were not more than 1.2%. When classifying the dimensions of qualitative features, the accuracy of the assessment was already somewhat lower. This fact could also be influenced by the processing software, which was not primarily intended for this technology. The biggest problem was with the resolution and dimensions of the overgrowth in the cross-section of the log, where the biggest difference between the real and scanned value was recorded. Manual evaluation in the software can also introduce parts of the subjective elements of the evaluator into the evaluation, so it may not be completely optimal.

Despite the detected differences, it was confirmed that CT technology used for scanning harvested wood can have a fundamental impact on the optimization procedures in the recovery and processing of wood. The disadvantage is that, in addition to high investment costs, it also requires experienced personnel consisting of operators who would evaluate obtained data. A simpler way of its possible use for forestry operations and smaller wood processing operations can be the partial rental of this equipment for a certain capacity of wood volume. In regional proximity, where locational rent does not play such an important role, this technology could become an important factor that would improve the economic evaluation of the final production in both sectors of the forestry–timber complex and thus confirm the fulfillment of the principles of the bioeconomy.

Renting a CT scanner can be important when organizing a wood auction. Valuable log cuts are offered in wood auctions. Using a CT scanner, it is possible to create a quality certificate for each piece of log with an overview of internal defects. Such information has a fundamental impact on buyers in terms of their willingness to buy the offered cutouts and thus on the success of the auction overall.

Other examples are the production of luxury wood products such as musical instruments, sports equipment, alcohol barrels, and gun stocks. For these products, high demands are placed on the quality of the raw material input. CT technology would enable optimal production with high added values, even for smaller enterprises. This technology is the most relevant and economically justified for assessing the quality of valuable or unique tree species.

## Figures and Tables

**Table 1:** Identification and dimensions of qualitative features based on visual assessment and measurement in accordance with STN EN 1309-3 and from CT scans of the beech log.

Feature	Cut Nr.	Measurement According to STN EN 1309-3		Measurement from CT Scans	
		Distance from the Log End (mm)	Feature Size (mm, % from End Surface Area)	Distance from the Log End (mm)	Feature Size (mm)
Knot + bark pocket	11	3300	220	3190	221
Knot	13	700	230	710	214.5
Knot + bark pocket	2	800	145	790	109
Knot	2	1500	145	1470	135
Knot	5	2460	140	2470	163
False heart	10	0	75%	no	no
Knot	3	1500	85	1490	85.5

**Table 2:** Identification and dimensions of qualitative features based on visual assessment and measurement in accordance with STN EN 1309-3 and from CT scans of the oak log.

Feature	Cut Nr.	Measurement According to STN EN 1309-3		Measurement from CT Scans	
		Distance from the Log End (mm)	Feature Size (mm, % from End Surface Area)	Distance from the Log End (mm)	Feature Size (mm)
Knot + bar pocket	3	810	60	808	98.5
Knot	3	1310	65	no	no
Knot + bark pocket	6	1600	50	1610	54
Knot	8	1110	30	1149	27
Knot	8	480	45	468	49
Bark pocket	8	920	20	916	85
Knot	5	410	30	413	44
Knot	5	1980	20	1988	33.5

**Table 3:** Evaluation of the differences between the manually measured dimensions of qualitative features and the dimensions detected with CT analysis on beech and oak logs.

Feature	Cut Nr.	Differences on Beech Log			
		Distance from the Log End	Difference in %	Feature Size (mm)	Difference in %
Knot + bark pocket	11	110	3.33	−1	−0.45
Knot	13	−10	−1.43	15.5	6.74
Knot + bark pocket	2	10	1.25	36	24.83
Knot	2	30	2.00	10	6.90
Knot	5	−10	−0.41	−23	−16.43
False heart	10				
Knot	3	10	0.67	−0.5	−0.59
**Average**			**0.90**		**3.5**
Knot + bar pocket	3	2	0.25	38.50	64.17
Knot	3	no	no	no	no
Knot + bark pocket	6	10	0.63	4.00	8.00
Knot	8	39	3.51	3.00	10.00
Knot	8	12	2.50	4.00	8.89
Bark pocket	8	4	0.43	65.00	325.00
Knot	5	3	0.73	14.00	46.67
Knot	5	8	0.40	13.50	67.50
**Average**			**1.21**		**75.75**

**Table 4:** Basic statistical characteristics of qualitative feature dimensions identified with manual measurement and through CT scanning.

	Real Detection	CT Detection
**N**	15	15
**Mean**	85.72	87.93
**Median**	60.00	85.00
**Modus**	Multiple	0.000000
**Minimum**	0.75	0.00
**Maximum**	230.00	221.00
**Variance**	5351.10	4920.28
**Std. Deviation**	73.15	70.14
**Variation coeff.**	85.34	79.77

## Data Availability

Data sharing not applicable.
